# Anaphylactic Bronchospasm during Induction of General Anaesthesia: A Case Report

**DOI:** 10.31729/jnma.4304

**Published:** 2019-08-31

**Authors:** Binod Gautam, Rashna Shakya

**Affiliations:** 1 Department of Anaesthesia and Intensive Care, Kathmandu Medical College Public Limited, Sinamangal, Kathmandu, Nepal

**Keywords:** *allergy*, *anaphylaxis*, *bronchial spasm*, *general anesthesia*

## Abstract

Bronchospasm represents the clinical manifestation of bronchial muscles contraction resulting in reduced alveolar air flow. Non-allergic mechanisms or anaphylaxis underlie the genesis of perioperative bronchospasm, a potential anaesthetic disaster. Early recognition and treatment are crucial. We report a rare incident of anaphylactic bronchospasm without hypotension during general anaesthesia. Urticaria appeared in chest and abdomen suggesting anaphylaxis. After the event resolved with bronchodilators, surgery continued uneventfully. Vecuronium was the most probable culprit but confirmation was not possible as the patient was lost to follow up. Rarely, perioperative anaphylaxis presents only with bronchospasm that requires prompt attention to avoid adverse outcome.

## INTRODUCTION

Bronchospasm occurring during anaesthesia is not unusual. It accounts for 3% of all intraoperative critical incidents.^1^ Non-allergic or allergic mechanisms underlie its origin. During general anaesthesia, bronchospasm is attributed to anaphylaxis in 14% of cases.^1^ Rarely, bronchospasm may be the sole presenting feature of anaphylaxis.^2^

In most cases bronchospasm resolves uneventfully. Regardless of genesis, it can however, cause hypoxia, hypotension and may prove fatal even when appropriately treated.^2^ Being rare but potentially grievous, we report an incident of anaphylactic bronchospasm with urticaria and without hypotension that occurred during induction of general anaesthesia in a young male undergoing laparoscopic cholecystectomy.

## CASE REPORT

A 36 years male (height 1.7 meters, weight 82 kilograms) having diagnosed with empyema gall bladder was being prepared for laparoscopic cholecystectomy. The patient was not receiving any chronic medications and had no prior anaesthetic exposure. He was a chronic cigarette smoker. There was no history suggestive of atopy. Physical examination and investigations were normal.

General anaesthesia was induced with intravenous Fentanyl 100 micrograms (mcg), Propofol 200 milligrams (mg) and gradually increasing concentration of inhaled Halothane. After confirming adequate mask ventilation Vecuronium eight mg was administered. At 90 seconds, tachycardia was noticed together with gradually decreasing oxygen saturation (SPO_2_). Three minutes past Vecuronium administration, tracheal intubation was performed with ease. Immediately, ventilation was not possible. Chest was silent on auscultation. Misplacement and occlusion of tracheal tube and breathing circuit were ruled out. With elevated airway pressure, SPO_2_ of 70% and unrecordable capnogram, severe bronchospasm was diagnosed. Instantaneously, Halothane (3%) was switched on and ten Salbutamol puffs (100 mcg/puff) were sprayed down tracheal tube. Lignocaine 100 mg and Ketamine 40 mg were injected intravenously. Gentle attempts at manual ventilation were continued. Salbutamol puffs were repeated three minutes later. At fifth minute of intubation, wide-spread wheezes were audible. SPO_2_ gradually improved with appearance of up-sloping capnograph.

Urticaria appeared in arms, chest and abdomen once the most critical moment resolved. Wheezes disappeared and normal intensity breath sounds were heard nine minutes after intubation. Tracheal suctioning revealed minimal mucoid secretions. Methylprednisolone 200 mg and Pheniramine Maleate 20 mg were administered. Hypotension was not observed.

With controlled cardio-respiratory parameters for next 10 minutes, it was decided to go ahead with surgery, which proved uneventful. Anaesthesia was maintained with Halothane in 100% oxygen. Minute ventilation was adjusted to maintain eucapnia employing tidal volume 400 milliliters and increased expiratory time.

Postoperative course was uneventful. Chest X-ray did not reveal acute changes. On discharge, patient was advised to undergo intra-dermal testing after six weeks to identify the offending agent, but he did not show up.

## DISCUSSION

The incident in our case highlights the rare entity of anaphylaxis presenting primarily with bronchospasm. Anaphylaxis during anaesthesia remains a major cause of concern. Rapidity and variation in its presentation makes diagnosis and management difficult. Hypotension is the most common feature of anaphylaxis during general anaesthesia.^1,2^ Although hypotension was lacking, appearance of skin changes and severity of bronchospasm made us believe that the incident in our case was of anaphylactic origin.^3^ In 20% of cases, anaphylaxis manifests with isolated sign, and, rarely bronchospasm is a sole feature.^2,4^

Perioperative bronchospasm is more frequently associated with non-allergic mechanisms in comparison to anaphylaxis.^1,2^ Upper respiratory tract infection, asthma, bronchitis and smoking include the most important risk factors.^1^ Tracheal irritants, aspiration, pulmonary edema, inadequate anaesthetic depth and histamine or serotonin release are also notably implicated.

Prevalence of anaphylaxis is estimated at 1/4000 to 1/25000 anaesthetic procedures, with neuromuscular blocking agents accounting for 58% of cases.^2^ The most likely cause in our case also was Vecuronium. It contains a quaternary ammonium ion which is the main allergenic structure of neuromuscular blocking agents.^5^ The temporal events leading to the incident in our case started seconds after Vecuronium administration. With the background of patient's smoking history, well-built body habitus, and absence of hypotension and urticaria at the moment of presentation, mechanical (intubationinduced) factor was initially thought to be responsible. Lack of chest auscultation before tracheal intubation, however, comprised the limitation in our part as we might have missed to recognize bronchospasm a minute earlier.

Other possibilities included Latex, Propofol and Fentanyl. Latex is the second most common cause for perioperative anaphylaxis.^2^ But it was less likely in our patient as latex-induced anaphylaxis mostly develops 30 minutes after surgical incision and usually involves cardiovascular compromise.^6^ Also, it typically occurs in patients with history of allergy which was not suggestive in our patient.^2,6^ Latter factors also make it less likely for Propofol being the culprit.^2,7^ Fentanylinduced anaphylaxis is very rare, primarily presents with hypotension and urticaria, whereas pulmonary involvement is unusual.^8^

Diagnosis of anaphylaxis might be retrospectively supported by raised serum Tryptase level.^2,3^ However, it solely based on clinical features in our case owing to unavailable facility. Causative agent for anaphylaxis is essentially identified with intra-dermal testing.^2,3^ But it was not possible as we lost our patient to follow up, which is not an unusual diagnostic challenge in our part of the world.

On suspecting bronchospasm, the priority is its reversal and to prevent hypoxia, hypotension and pneumothorax irrespective of underlying cause. Mainstay of management includes use of 100% oxygen, hand ventilation, and bronchodilators and avoidance of patient stimulations. Salbutamol puffs are the first line bronchodilators. Its repeated dose and high concentration of Halothane, together with Lignocaine and Ketamine were effective in our patient. Salbutamol can also be given intravenously. Epinephrine is advised when cardiovascular compromise is associated.^4,6^ Magnesium, inhalational anaesthetics and Ketamine comprise the second line bronchodilators.^4^ Glucocorticoids and antihistamines may be considered once patient becomes stable.^4,6^

Anaphylaxis during general anaesthesia can present with varying severity of bronchospasm and without hypotension. Any anaesthetic medication might be responsible. Management of perioperative bronchospasm must aim to secure airway, establish alveolar air flow and prevent complications. Reporting of critical incidents and preserving records is essential for the further reference. Educating patients and family regarding these rare but potentially fatal drug reactions and identifying the offending agent is central to prevent untoward event in the future.

## Consent:

**JNMA Case Report Consent Form** was signed by the patient and the original is attached with the patient's chart.

## Consent:

**None.**

## References

[1] Westhorpe RN, Ludbrook GL, Helps SC (2005). Crisis management during anaesthesia: bronchospasm. QualSaf Health Care.

[2] Mertes PM, Alla F, Trechot P, Auroy Y, Jougla E (2011). Grouped'Etudes des Reactions AnaphylactoidesPeranesthesiques. J Allergy ClinImmunol.

[3] Fisher MM, Ramakrishnan N, Doig G, Rose M, Baldo B (2009). The investigation of bronchospasm during induction of anaesthesia. ActaAnaesthesiol Scand.

[4] Kolawole H, Marshall SD, Crilly H, Kerridge R, Roessler P (2017). Australian and New Zealand Anaesthetic Allergy Group/Australian and New Zealand College of Anaesthetists Perioperative Anaphylaxis Management Guidelines. Anaesth Intensive Care.

[5] Baldo BA, Fisher MM, Pham NH (2009). On the origin and specificity of antibodies to neuromuscular blocking (muscle relaxant) drugs: an immunochemical perspective. Clin Exp Allergy.

[6] Dewachter P, Mouton-Faivre C, Emala CW (2009). Anaphylaxis and anesthesia: controversies and new insights. Anesthesiology.

[7] Nishiyama T, Hanaoka K (2001). Propofol-induced bronchoconstriction: two case reports. Anesth Analg.

[8] Bennett MJ, Anderson LK, McMillan JC, Ebertz JM, Hanifin JM, Hirshman CA (1986). Anaphylactic reaction during anaesthesia associated with positive intradermal skin test to fentanyl. Can Anaesth Soc J.

